# Serosurveillance of *Brucella* antibody in food animals and role of slaughterhouse workers in spread of *Brucella* infection in Southeast Nigeria

**DOI:** 10.14202/vetworld.2018.1171-1178

**Published:** 2018-08-27

**Authors:** Samuel Okezie Ekere, Emmanuel Okechukwu Njoga, Joseph Ikechukwu Onunkwo, Ugochinyere Juliet Njoga

**Affiliations:** 1 Department of Veterinary Obstetrics and Reproductive Diseases, Faculty of Veterinary Medicine, University of Nigeria, Nsukka, Enugu State, Nigeria; 2 Department of Veterinary Public Health and Preventive Medicine, Faculty of Veterinary Medicine, University of Nigeria, Nsukka, Enugu State, Nigeria

**Keywords:** *Brucella* antibody, brucellosis, cattle, goats, slaughterhouse workers

## Abstract

**Aim:**

The study was carried out to determine the seroprevalence of *Brucella* antibody in slaughter cattle and goats; and the role of slaughterhouse workers (SHWs) in spread of *Brucella* infection during slaughterhouse operations in Enugu State, Southeast Nigeria.

**Materials and Methods:**

Rose Bengal plate test was used to screen for *Brucella* antibody in 484 cattle and 340 goats slaughtered for human consumption in the state. Structured and pretested questionnaire was used to elicit information from randomly selected SHWs, on socioeconomic characteristics, awareness of brucellosis and involvement in practices that aid dissemination of *Brucella* infection during slaughterhouse operations.

**Results:**

Suspected seroprevalence of 2.5% and 4.1% were recorded for *Brucella* antibody in cattle and goats respectively. There was poor awareness of brucellosis (32.1%) among the workers surveyed. Slaughterhouse practices that aid acquisition or spread of *Brucella* infection and percentage of SHWs engaged in the practices are: non-use of personal protective clothing during slaughterhouse operations (70.8%), discharge of eviscerated fetuses or pregnant uterine contents by open-air dump method of refuse disposal (64.9%) and illegal sell of eviscerated fetuses or gravid uterine contents for human consumption (59.9%) or preparation of dog food (71.5%).

**Conclusion:**

The 4.1% suspected seroprevalence of *Brucella* antibodies in goats represents 128% increase from 1.8% seroprevalence earlier reported in the same species and study area in 2009. Significant amounts of *Brucella* antibody was detected in the food animals screened. Slaughterhouse workers played significant roles in spread of *Brucella* infection by their involvement in risk practices and behaviours that facilitate pathogen transmission. Therefore, massive awareness campaign and coordinated brucellosis control program in Enugu State are imperative to forestall the zoonotic and economic consequences associated with brucellosis.

## Introduction

Livestock production is a major employer of labor in most developing economies. In agrarian communities in Sub-Saharan Africa, livestock production is widely practiced due to the availability of abundant lush pasture at no cost to the farmers; and also as precautionary measure against crop failure. In most traditional African settings, ownership of livestock is a measure of economic and social status; as well as a form of cash reserve for financing immediate family needs. However, the endemicity of microbial pathogens, such as *Brucella* in tropical climatic regions, has been a major drawback to livestock farming and the profitability of the farming business in these parts of the world.

Brucellosis is a dreaded bacterial zoonosis with great public health and food safety importance. *Brucella* pathogens in edible animal tissues are transmissible to humans through the food chain and human food habits, once formed are very difficult to change. The disease has a cosmopolitan distribution, and affects economically important domestic livestock as well as a wide range of other terrestrial and aquatic animals [1,2]. Although more than eight different *Brucella* species have been described, *Brucella abortus*, *Brucella melitensis* and *Brucella suis* are responsible for most of the disease burden globally [3]; preferentially infecting cattle, small ruminants and swine respectively. Despite their distinct host preferences, *Brucella* agents cause brucellosis of varying severity in most terrestrial animals and humans [4]; especially in mixed husbandry systems or at the animal-human interfaces [3].

The economic importance of brucellosis in livestock production finds expression in reduced productivity in livestock due to infertility problems caused by the disease agents [5], high costs of treatment, prevention or control measures against the disease [6], and heavy financial losses occasioned by restrictions in local and international trades in infected animals or their products [7]. Brisibe *et al*. [8] estimated an annual loss of US$3.2 million to brucellosis in only two States in Nigeria while Bamaiyi *et al*. [9] reported a loss of US$2.6 million per annum in Malaysia due to the disease.

Brucellosis occurs in most food-producing animals and may be acquired venerally, congenitally, through inhalation of aerosolized *Brucella* organisms in overcrowded or overstocked settings [3-6]. The disease in animal may also spread by contact with or ingestion of fluids or tissues from infected animals [2,10]. *B. melitensis* is by far the most virulent *Brucella* organism and the genus with the highest zoonotic potential [11]. The organism has a very low infective dose of just about 10 organisms [12, 13], capable of penetrating a host through skin abrasion [14] and causing brucellosis in a broad host range (goats, sheep, cattle and humans) under natural conditions [6,14]. Infertility problems such as middle or late-term abortion, birth of weak/unthrifty neonates and repeat breeder syndrome are major clinical manifestations of brucellosis in food animals leading to mass or frequent culling of infected animals for slaughter.

In most developing countries in the tropics, food animals are mostly slaughtered at homes, slaughterhouses/slabs or at clandestine locations due to a limited number of standard abattoirs. At these slaughter points, factors such as tropical climatic conditions, unsafe hygienic practices among slaughterhouse workers and inadequate knowledge of brucellosis as well as the dynamics of the disease spread tend to facilitate *Brucella* transmission from food animals to humans. These factors seem to also favor the persistence of *Brucella* pathogens in the environment and its onward transmission to animals grazing or scavenging around the slaughter points and meat processing environment. Food animals and pets are reservoirs of human brucellosis and high brucellosis burden in animals is a major determinant of the human disease [15,16]. Brucellosis outbreaks in livestock populations are usually triggered by poor farm management practices which permit the use of infected animals for breeding or defective biosecurity programs in farms where “farm to fork” concept of food safety is largely ignored.

The incidence of human brucellosis is about 500,000 cases per year worldwide [14, 17] and is usually caused by *B. abortus, B. melitensis, B. suis* or *B. canis* [13]. Although *B. ovis* is the principal agent of ovine brucellosis, the agent has not been associated with any case of human brucellosis [13]. *B. melitensis* and *B. abortus* are the most important and most frequent causes of human brucellosis often implicated in most cases of the human disease worldwide [12,18]. Human brucellosis is a disease of variable manifestations, with severe debilitating complications that warrant prolonged therapy with antibiotic combinations. Although there is no vaccine against human brucellosis, human-to-human transmission of the disease has not been reported [2,14]. Effective control of human brucellosis transmission via the food chain depends on strict compliance to food safety measures such as milk pasteurization and proper cooking of animal product before consumption.

Brucellosis poses a serious public health threat to animal health workers, livestock farmers and slaughterhouse workers (SHWs), who are particularly at risk of the disease due to their occupational exposure. Animals are important source of *Brucella* infection to humans and the risk of human exposure to brucellosis depends on the disease burden in animals [19]. Brucellosis in humans may be acquired through consumption of infected raw or undercooked animal products, as well as by wound contamination with infected fluids or tissue [14]. Factors that may affect human exposure to brucellosis, particularly among the occupationally exposed individuals, include non-use of personal protective clothing (PPC) during routine operations and inadequate knowledge of the disease and its transmission dynamics [20].

Although brucellosis has been controlled in most industrialized nations, the disease has become a neglected zoonosis in some tropical or developing countries due to lack of sustainability in the disease prevention and control programs [10,12]. Consequently, brucellosis has continued to ravage these parts of the world, where livestock farming is coincidentally a major employer of labor and source of livelihood [11,14].

Establishment of adequate control programs against brucellosis in a population depends on the presumptive diagnosis of the infection. Diagnosis of brucellosis by culture and isolation of *Brucella* organisms from clinical samples is the preferred method of diagnosis, but this method is laborious, time-consuming, risky and its outcome depends on the competence of the laboratory personnel [21]. Serological tests offer best alternatives to culture and isolation method of diagnosis since the tests are easy to perform, less risky and provide result within a short period. Rose Bengal plate test (RBPT) has been recommended for brucellosis screening due to its high sensitivity and relatively low cost, especially in developing countries where the disease burden may be high and the facilities for modern methods of diagnosis unavailable [2].

Despite many reports on the occurrence of *Brucella* antibody in cattle [16,19,20,22,23] and goats [19,22,24] in other parts of Nigeria; data on the seroprevalence of *Brucella* antibody in food animals in the study area are dated, few and far between. Information on the role of SHWs on slaughterhouse practices that facilitate dissemination of *Brucella* infections during slaughterhouse operations in Enugu State is lacking.

The study was therefore carried out to determine the seroprevalence of *Brucella* antibody in slaughter cattle and goats; and also the role of SHWs in spread of *Brucella* infection, during routine slaughterhouse operations in Enugu State.

## Materials and Methods

### Ethical approval

Ethical clearance for care and use of animals is not applicable to this study since blood samples used were collected from slaughtered animals at the abattoir.

### Informed consents

Oral consent to participate in the study was sought and obtained from all human subjects included in the study. Consequently, 137 respondents were randomly selected and interviewed from those who consented to participate in the survey.

### Study area

This study was carried out in Enugu State, Southeast Nigeria. The state has map coordinates of 6^0^30’ North$$$ and 7^0^30’ East and a population of about 5 million people [25]. Enugu State has a tropical climate characterized by wet (April to October) and dry (November to March) seasons. The state is typically an agrarian society dominated by crop farmers and civil servants. However, small and medium scale food animal production, as additional source of income or precautionary measure against crop failure is widely practiced.

### Questionnaire survey

Structured and pretested questionnaire (closed ended) was used to extract information on educational level, awareness of brucellosis, use of PPC during routine slaughterhouse operations and method of disposal of slaughterhouse waste from 137 randomly selected SHWs. The questionnaire was administered in the form of an interview, in indigenous language, to respondents who are limited in their ability to read or write English language. Thereafter, the questionnaires were retrieved and the responses collated, analyzed and presented in tables.

### Sample collection

Research visits to three major slaughterhouses (Ikpa, Akwata and 9^th^ mile) in Enugu State for blood sample collection were made weekly for 6 months; consisting of 3 months of dry season (January-March) and another 3 months of rainy season (June-August). The simple random sampling method was used to select animals to be sampled. The sex and breed of each selected animal were determined by visual examination while age was estimated using history (if available) and teeth eruption and wear method as described by Pace and Wakeman [26]. Cattle and goats that are <1 year old do not usually present signs of brucellosis or are slaughter for food, and were therefore excluded from the study. About 5-10 mL of blood was aseptically collected from each selected animal during bleeding. The blood samples were kept in a slant position for about 3 h and sera samples formed were harvested and stored at −20°C until the brucellosis screening test was performed.

### Sample screening

The RBPT as described by Amin *et al*. [27] was performed by mixing equal volumes (30 μl) of stained *Brucella* antigen and test serum thoroughly on a clean glass plate, and then followed by gentle stirring of the plate for about 4 min using an applicator stick. *B. abortus* and *B. melitensis* antigens were used to screen sera samples from cattle and goats respectively. The antigens were procured from the Veterinary Laboratory Agency, Addle stone, Surrey, KT 15-3NB United Kingdom, and preserved at −20°C. Sera samples that formed distinct granules (agglutination) within 4 min of stirring were recorded as positive, containing detectable amount of *Brucella* antibody; while samples that formed no granules were considered negative, containing no or undetectable amount of the antibody.

### Statistical analysis

Chi-square statistic was used to test for significant association (p<0.05) between *Brucella* seropositivity and species, breed, sex, age and season. The statistic was also used to check for significant association (p<0.05) between awareness of brucellosis and demographics and socioeconomic characteristics of the respondents. In addition, statistically significant associations between slaughterhouse practices and educational levels and demographics were also tested for at p<0.05. All the tests were done using IBM^®^ SPSS statistics version 23 (SPSS Inc., Chicago, Illinois) at 5% probability level.

## Results

Suspected seroprevalence values of 2.5% and 4.1% were recorded for *Brucella* antibody in cattle and goats respectively (Table-1). There was no significant association found at p = 0.349 between *Brucella* seropositivity and the species; although the odds of the disease were about 2 times higher (odds ratio =1.7, 95% confidence interval: 0.557 - 5.118) in goats than cattle.

**Table-1:** Seroprevalence of *Brucella* antibody in cattle and goats slaughtered in Enugu State, Nigeria.

Species	Number screened	Number positive	Prevalence	Odds ratio	95% CI	χ^2^ value	p-value
Goats	340	14	4.1	1.69	0.5575.118	0.877	0.349
Cattle	484	12	2.5				
Total	824	26	3.2				

CI: Confidence interval

The results on breed, sex, age and seasonal distribution of *Brucella* antibody in cattle and goats are presented in Tables-2 and 3 respectively. *Brucella* antibody was detected mostly in adult and old animals in both cattle (Table-2) and goats (Table-3), but no significant association (p<0.05) was found between the occurrence of *Brucella* antibody and breed, age and season in both species. However, sex was strongly associated with *Brucella* seropositivity in cattle at p = 0.02.

**Table-2:** Breed, sex, age and seasonal distribution of *Brucella* antibody in cattle (n=484) surveyed in Enugu State, Nigeria.

Variables	Number tested	Number positive	Prevalence	χ^2^ value	p-value
Breed					
White fulani	286	6	2.1	1.556	0.459
Sokotogudali	172	4	2.3		
Red bororo	26	2	7.7		
Sex					
Cow	178	9	5.1	7.786	0.02*
Bull	306	3	0.98		
Age (years)					
Young (1-3)	76	2	2.6	0.37	0.858
Adult (3-8)	290	6	2.11		
Old (>8)	118	4	3.4		
Season					
Wet (Winter)	168	4	2.38	0.05	0.943
Dry (Summer)	316	8	2.53		

* Denotes statistically significant P value, Chi-square statistic

**Table-3:** Breed, sex, age and seasonal distribution of *Brucella* antibody in goats (n=340) surveyed in Enugu State, Nigeria.

Variables	Number tested	Number positive	Prevalence	χ^2^ value	p-value
Breed					
Kano brown	244	8	3.3	1.574	0.455
Sokoto red	82	5	6.1		
Sahel	14	1	7.2		
Sex					
Buck	58	6	10.3	3.43	0.06
Doe	282	8	2.8		
Age (years)					
Young (12)	48	2	2.3	1.06	0.59
Adult (26)	196	6	3.7		
Old (>6)	96	6	3.8		
Season					
Wet (Winter)	158	6	3.8	0.38	0.85
Dry (Summer)	182	8	4.4		

The majority (67.9%) of the SHWs had not heard of brucellosis (Table-4). The workers were massively involved in practices that could aggravate spread of *Brucella* infection such as non-use of PPC during slaughterhouse operations (70.8%), disposal of slaughterhouse wastes, including eviscerated fetuses and pregnant uterine contents, by open-air dump method (64.9%) and illegal sale of eviscerated fetuses for human consumption (59.9%) or preparation of dog food (71.5%). There was no significant association (p>0.05) between awareness of brucellosis and gender and age of the SHWs; but statistically significant association was found between educational levels of the respondents and knowledge of brucellosis at p = 0.004 (Table-5). Similarly, significant associations (p<0.05) was found between educational levels of SHWs and use of PPC (Table-6) and sell of eviscerated fetuses for human consumption (Table-7).

**Table-4:** Awareness of brucellosis and slaughterhouse practices among slaughterhouse workers (n=137) surveyed in Enugu State, Nigeria.

Information required	Number of respondents (%)
Have heard of brucellosis	
Yes	44 (32.1)
No	93 (67.9)
Use of PPC while on duty	
Yes	40 (29.2)
No	97 (70.8)
Regularity of the use of PPC among “yes” respondents	
Always	11 (27.5)
Sometimes	17 (42.5)
Seldom	12 (30)
Reason for non-use of PPC among “no” respondents	
Perceived inconvenience	71 (73.2)
Non-availability of PPC	9 (9.3)
High cost of PPC	2 (2.2)
No response	16 (16.5)
Have experienced undulating fever that coincided with abortion or orchitis	
Yes	18 (13.1)
No	119 (86.9)
Practiced open-air dump method of disposing eviscerated fetuses	
Yes	89 (64.9)
No	21 (15.3)
No response	27 (19.7)
Sold fetuses harvested during evisceration for human consumption	
Yes	82 (59.9)
No	31 (22.6)
No responses	24 (17.5)
Sold fetuses harvested during evisceration for preparation of dog food	
Yes	98 (71.5)
No	18 (13.1)
No response	21 (15.3)

PPC=Personal protective clothing

**Table-5:** Association between awareness of brucellosis, demographics and educational levels of SHWs (n=137) surveyed in Enugu State, Nigeria.

Demographic variables	Number of SHWs (%)	Number of SHWs who have heard of brucellosis	χ^2^ value	p-value
Gender				
Male	103 (75.2)	37	2.757	0.097
Female	34 (24.8)	7		
Age (Years)				
<30	14 (10.2)	10	10.033	0.007
30-40	41 (29.9)	12		
41-50	44 (32.1)	11		
51-60	28 (20.4)	6		
>60	10 (7.3)	5		
Highest educational level attained				
No formal education	14 (10.2)	5	13.324	0.004*
Primary	64 (46.8)	11		
Secondary	42 (30.7)	19		
Tertiary	17 (12.4)	9		

* Denotes statistically significantP values, SHWs=Slaughterhouse workers

**Table-6:** Association between slaughterhouse practices and demographics and educational levels of SHWs (n=137) surveyed in Enugu State, Nigeria.

Demographic variables	Number of respondents (%)	Number of SHWs that used PPC	*χ*^2^ value	p-value	Number of SHWs that disposed fetuses by openair dump method	*χ*^2^ value	p-value
Gender							
Male	103 (75.2)	33	1.621	0.203	69	7.13	0.008
Female	34 (24.8)	7			20		
Age (Years)							
<30	14 (10.2)	7	7.782	0.1	11	4.81	0.308
30-40	41 (29.9)	13			22		
41-50	44 (32.1)	10			29		
51-60	28 (20.4)	5			21		
>60	10 (7.3)	5			6		
Highest educational level attained							
No formal education	14 (10.2)	5	20.89	0.000*	10	10.19	0.017*
Primary	64 (46.8)	8			49		
Secondary	42 (30.7)	16			23		
Tertiary	17 (12.4)	11			7		

* Denotes statistically significantP values, SHWs=Slaughterhouse workers, PPC=Personal protective clothing

**Table-7:** Association between the method of disposal of eviscerated fetuses and demographics and educational levels of SHWs (n=137) surveyed in Enugu State, Nigeria.

Demographic factors	Number of respondents (%)	Sold fetuses for human consumption	*χ*^2^ value	p-value	Sold fetuses for preparation of dog food	*χ*^2^ value	p-value
Gender							
Male	103 (75.2)	61	0.69	0.793	67	0.069	0.079
Female	34 (24.8)	21			31		
Age (Years)							
<30	14 (10.2)	8	17.40	0.002*	10	23.46	0.000*
30-40	41 (29.9)	21			31		
41-50	44 (32.1)	37			31		
51-60	28 (20.4)	11			20		
>60	10 (7.3)	5			7		
Highest educational level attained							
No formal education	14 (10.2)	13	17.63	0.001*	11	9.189	0.027*
Primary	64 (46.8)	44			47		
Secondary	42 (30.7)	20			33		
Tertiary	17 (12.4)	5			7		

* Denotes statistically significantP values, SHWs=Slaughterhouse workers

## Discussion

The suspected seroprevalence of 4.1% and 2.5% recorded for *Brucella* antibody in goats and cattle respectively in this study are lower than the findings of Junaidu *et al*. [22]; who reported seroprevalence of 20.76% and 32.2% respectively for goats and cattle in northern Nigeria. At the international level, particularly in Ethiopia and India, our findings are also lower compared to 9.86% in goat and 11.74% in cattle as reported by Negash *et al*. [28] and Kaushik *et al*. [29] respectively.

The disparity in the seroprevalence of *Brucella* antibody found in this and the other studies in various study areas, could be attributed to discrepancies in epidemiological factors capable of influencing the disease dynamics. These factors include livestock husbandry practices, extent of pasture or pastureland contamination with *Brucella* agents, climatic conditions, source of breeders or replacement stocks, individual differences in interpretation of screening test results and total number of animals sampled.

The herding of different animals species together and practice of extensive husbandry system of livestock production are important factors in the epidemiology of dissemination of *Brucella* infection in animal populations. These practices favor contamination of pasture and pastureland with *Brucella* agents [19] and facilitate exchange of *Brucella* species between animals. In Enugu State, cattle and goats are usually not herded together and extensive (free range) husbandry practice is very rare. This may have accounted for the low suspected seroprevalence being reported.

Despite the low seroprevalence found in this work, the threat of brucellosis in the study area still subsists as a single infected animal or individual can easily spread the disease within or across the population. The suspected seroprevalence of 4.1% in goat found in this study is higher than 1.8% earlier reported by Onunkwo *et al*. [30] in the same species and study area. This 128% increase in *Brucella* antibody in goats in less than a decade, clearly shows that caprine *Brucella* infection has continued to rise unabatedly; and there is an impending danger of brucellosis outbreak and its associated public health and economic consequences in the study area.

Higher seroprevalence of *Brucella* antibody in goats than cattle may be attributed to the fact that there is no coordinated national brucellosis control programs in goats in Nigeria [24]. Vaccination and other control programs instituted against brucellosis in the past target just the cattle population; not minding the fact that other non-targeted animal species can become reservoirs of the infection for transmission to human and animals or re-infection of even the targeted species. This underscores the need for mass brucellosis vaccination campaign, targeting all susceptible animal species, using Rev. 1 or S19 *Brucella* strains.This may provide a better result in animal brucellosis control in the country that the current practice that targets only cattle with S19 strain. In addition, the low grazing feeding habit and voracious or “catholic” appetite of goats; unlike cattle, may increase their propensity for *Brucella* infection, especially when grazing on contaminated pastureland. This further explains the higher *Brucella* seropositivity found in goats than in cattle in this study.

The preponderance of *Brucella* antibody in females in both species could be attributed to the affinity which *Brucella* species have for female reproductive tract and fetal tissues; due to the production of erythritol, a 4-carbon sugar in these tissues that stimulate the growth of *Brucella* organisms [31]. In addition, female animals are generally kept for longer period in the farm than the males. The extended period of stay of female animals in the farm exposes them to *Brucella* organisms and hence the chances of acquiring the infection much more than the males. Furthermore, stress associated with pregnancy, parturition and lactation, which female animals usually undergo, tends to lower their immunity and predisposes them to infections with agents like *Brucella* species.

Although the presence of *Brucella* antibody in the SHWs was not determined due to uncooperative attitude of the workers; reports of undulating fever that coincided with abortion and orchitis in 13% of the respondents, is strongly evocative of existing *Brucella* infections. This is most probably in view of the workers involved in certain slaughterhouse practices that predispose to the infection during slaughterhouse operations.

Non-use of PPC by over 70% of SHWs surveyed suggests that the workers are not just at risk of *Brucella* infection but represent an important epidemiological link in the transmission of the infection from animals to humans. The animal to human transmission of *Brucella* infection in Enugu State may be worsened by the fact that most ruminants being slaughtered in the state are sourced from the northern parts of the country, where very high *Brucella* antibody levels of 37% in cattle [23] and 30.76% in goat [22] have been reported. An effective control to this important epidemiological link in transmission of brucellosis from animal to human is provision of free PPC to SHWs for compulsory use during their routine operations and imposition of severe sanctions against defaulters, to tackle the problem of non-use of PPC due to perceived inconveniences.

Improper disposal of slaughterhouse waste, including eviscerated fetuses and other uterine contents by open-air dump method and sale of eviscerated fetuses or pregnant uterine contents for human consumption, as evidenced in this study are potent means of spreading *Brucella* infection. Open air dump method of disposal of pregnant uterine contents favors the contamination of pasture and pastureland with *Brucella* species and other infectious agents. This makes acquisition of *Brucella* infection almost inevitable for animals gracing or scavenging around refuse dumps and carcass processing areas. The sale of fetuses or pregnant uterine content for human consumption in the name of “cheap meat” predisposes buyers to *Brucella* infection, especially during handling and processing of the meat.

In view of the low infective dose of *B. melitensis*, estimated at 10 - 100 colony-forming units [12,13,31,32], their potential for aerosol dissemination [31] and the ability of the organisms to cause protracted and incapacitating disease of enormous public health and economic consequences [12,14]; there is need for a coordinated brucellosis prevention and control programs in the study area to limit the spread of *Brucella* infection. Such programs must include awareness creation on the mode of acquisition or spread of *Brucella* infection, as well as the public health and economic consequences associated with the disease. The prevention or control program must, among other things, include mass vaccination campaign, provision of free PPC to occupationally exposed individuals for use during routine operation. The program should also include periodic and serological surveillance studies to monitor the antibody levels in animal and human populations and evaluate the success of the vaccination program respectively. The program should also make provisions for incinerators at the slaughterhouses for proper disposal of eviscerated fetuses and other slaughterhouse or biomedical wastes capable of transmitting *Brucella* or other infectious agents.

## Conclusion

This study has revealed suspected seroprevalence of 4.1% and 2.5% for *Brucella* antibody in goats and cattle respectively in Enugu State, Nigeria. The 4.1% seroprevalence in goats represents 128% increase from 1.8% seroprevalence recorded by Onunkwo *et al*. [30] in the same study area. This clearly shows that *Brucella* infection in goats is on the rise and there is an impending danger of brucellosis outbreak with its associated public health and economic consequences in the study area. SHWs played active roles in spread of *Brucella* infection as evidenced in non-use of protective clothing during slaughterhouse operations, discharge of eviscerated fetuses or uterine contents by open-air dump method of refuse disposal and sell of fetuses or pregnant uterine contents for human consumption. There is need for coordinated brucellosis prevention or control program, using One Health approach, to tame the tide of rising *Brucella* infection in Enugu State, for sustainable livestock production and to safeguard human health.

## Authors’ Contributions

JIO conceived and designed the study. SOE collected serum samples and screened them for *Brucella* antibodies. UJN designed the questionnaire and carried out the survey alongside SOE. EON and UJN carried out the statistical analysis. EON drafted and edited the manuscript. All authors read and approved the final manuscript.
